# Stable DNA Aptamer–Metal–Organic Framework as Horseradish Peroxidase Mimic for Ultra-Sensitive Detection of Carcinoembryonic Antigen in Serum

**DOI:** 10.3390/gels7040181

**Published:** 2021-10-25

**Authors:** Lingjun Sha, Mingcong Zhu, Fuqing Lin, Xiaomeng Yu, Langjian Dong, Licheng Wu, Rong Ding, Shuai Wu, Jingjing Xu

**Affiliations:** 1 Center for Molecular Recognition and Biosensing, School of Life Sciences, Shanghai University, Shanghai 200444, China; lingjun_sha21@163.com (L.S.); xiaomeng_yu2021@163.com (X.Y.); langjian_dong@163.com (L.D.); 2 Sino-European School of Technology of Shanghai University, Shanghai University, Shanghai 200444, China; cytheriacc@126.com (M.Z.); wulich1997@gmail.com (L.W.); Rongding_2021@163.com (R.D.); 3 School of Basic Medical Sciences, Fudan University, Shanghai 200433, China; 2030150160@fdu.edu.cn; 4 State Key Laboratory of Pharmaceutical Biotechnology, School of Life Sciences, Nanjing University, Nanjing 210023, China

**Keywords:** colloid nanorods, DNA tetrahedron nanostructure, catalytic oxidation, carcinoembryonic antigen, colorimetric detection

## Abstract

Carcinoembryonic antigen (CEA) is an important broad-spectrum tumor marker. For CEA detection, a novel type of metal–organic framework (MOF) was prepared by grafting CEA aptamer-incorporated DNA tetrahedral (TDN) nanostructures into PCN-222 (Fe)-based MOF (referred as CEA_apt_-TDN-MOF colloid nanorods). The synthesized CEA_apt_-TDN-MOF is a very stable detection system due to the vertex phosphorylated TDN structure at the interface, possessing a one-year shelf-life. Moreover, it exhibits a significant horseradish peroxidase mimicking activity due to the iron porphyrin ring, which leads to a colorimetric reaction upon binding toward antibody-captured CEA. Using this method, we successfully achieved the highly specific and ultra-sensitive detection of CEA with a limit of detection as low as 3.3 pg/mL. In addition, this method can detect and analyze the target proteins in clinical serum samples, effectively identify the difference between normal individuals and patients with colon cancer, and provide a new method for the clinical diagnosis of tumors, demonstrating a great application potential.

## 1. Introduction

The metal–organic framework (MOF) has been widely used in the fields of catalysis, separation, and sensing due to its unique crystalline porosity, flexible tailorability, and large specific surface area. Post-synthetic modification of MOF is mainly used to adjust the physical and chemical properties of the porous framework structure, so as to endow the MOF with specific functions for a variety of application fields [1,2]. Especially in the biosensor field, the development of biomolecule-functionalized MOF-based colloid nanoparticles is a research hotspot [3,4]. Biomolecule functionalization can effectively improve the stability and biocompatibility of MOF, resulting in significant performance even in a complex biological environment [5,6,7]. In addition, a large number of biomolecules can be designed based on the specific molecular recognition principle, such as aptamer technology [8] and peptide phage display technology [9], which can endow MOFs with specific recognition ability, thus leading to enhanced sensing performance. In principle, owing to the incorporated large amounts of organic ligands and the large specific surface area, it is easier to achieve functionalized modifications of MOF by biomolecules with respect to traditional inorganic materials [10,11]. At present, some research groups have reported some related work on functionalization technologies to modify the surface of MOF, so as to exhibit innovative biological functions. Typically, they mainly utilized chemically active groups such as amino, carboxyl, as well as azide groups of the organic ligands in MOF to achieve covalent cross-linking toward the biomolecules [12]. Although the functional modification of nucleic acids [13,14,15], peptide sequences [16,17], and antibodies [18] can be achieved via the traditional method, the real applications always suffer from being time-consuming, loss of bioactivity, and low loading capacity. These limitations put forward new requirements for the innovation of the construction technology.

To overcome the aforementioned limitations, Wang et al. recently reported a facile and efficient post-modification strategy by using the strong interaction between the phosphate group of dihydroxyphenylalanine (DOPA) and the metal site of zirconium MOF. The nano-MOFs crystals can be dispersed in various chemical solvents in the form of colloids [19]. Based on this strategy, they fabricated functional complexes of DNA-MOF that exhibited very great application potential in drug and protein delivery [20,21]. The vast majority of DNA sequences strictly abide by the Watson–Crick complementary pairing principle and have high stability and high polymer recognition ability. Thus, the designability of single-stranded DNA aptamers has given birth to an ideal aptamer material for modifying nano-MOFs [22,23], making it possible to modify molecular recognition elements on the MOFs’ interface for biosensors construction. In the process of interface assembly, single-stranded DNA is difficult to form an assembly layer due to its flexible structure, which easily affects its molecular recognition efficiency. DNA nanostructures have extremely high biocompatibility and stability in biological solutions. At the same time, the strict base-pairing principle makes it possible to integrate functionalized nucleic acids into nanostructures through rational design. Thus, DNA nanostructures have attracted much attention in biological fields such as bionic systems, medical diagnosis, and treatment [24,25,26]. Therefore, the use of rigid DNA nanostructures to assemble functional MOF materials provides an idea for the establishment of a new type of biosensing system. 

**Scheme 1:**
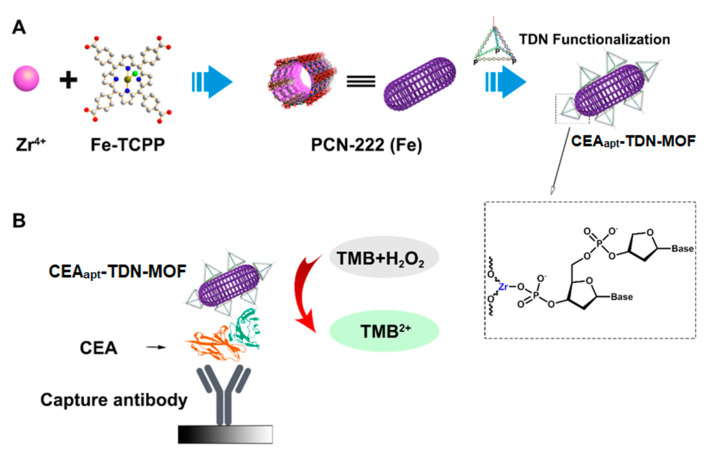
(**A**) Schematic diagram of the preparation process of CEA_apt_-TDN-MOF. First, Zirconia dichloride octahydrate and meso-tetra (4-carboxyphenyl) porphine ferric chloride (Fe-TCPP) were mixed to fabricate PCN-222 (Fe)-based metal–organic framework (MOF). Then, the CEA_apt_-TDN-MOF colloid nanorods were prepared by grafting carcinoembryonic antigen (CEA) aptamer-incorporated DNA tetrahedral (TDN) nanostructures. (**B**) Schematic diagram of the principle of constructing a colorimetric immunosensor for detecting CEA based on the CEA_apt_-TDN-MOF, where 3,3′,5,5′-tetramethylbenzidine (TMB) was used as the substrate.

In this work, we designed and prepared DNA tetrahedron nanostructure (TDN)-functionalized MOF colloid nanorods as a colorimetric biosensor for the sensitive detection of the tumor marker carcinoembryonic antigen (CEA) (Scheme 1, referred as CEA_apt_-TDN-MOF). Specifically, in order to achieve a good molecular recognition and interface assembly process, zirconium metal ions were used as ligands so as to realize high-efficiency coordination assembly with vertex phosphorylation-modified DNA tetrahedron, as well as the successful integration of a CEA aptamer sequence in a TDN structure for capturing targets. In addition, to achieve signal amplification, MOFs were endowed with very strong horseradish peroxidase mimicking activity by using iron tetrabenzoate porphyria. Meanwhile, a proteoglycan protein CEA, one of the most frequently detected tumor markers for early diagnosis of colon cancer, was chosen as our target [27]. As a result, the CEA aptamer sequences in TDN structures exhibited higher stability and molecular recognition properties than those single strands. Moreover, the tetrahedron nanostructure provided more accessibility toward the target by decreasing steric hindrance, which greatly improves the detection efficacy. Therefore, when CEA was captured by its antibody, our CEA_apt_-TDN-MOF could efficiently detect it by exhibiting a significant colorimetic signal. 

## 2. Results and Discussion

### 2.1. Preparation and Characterization of CEA_apt_-TDN-MOF Colloid Nanorods

As shown in Scheme 1, a nano-sized MOF material PCN-222 (Fe) was synthesized by using tetravalent zirconium ion and porphyrin ring Fe-TCPP as the precursor material. This PCN-222 (Fe) could exhibit very good structural stability, excellent biocompatibility, and horseradish peroxidase-like activity due to the presence of an iron porphyrin ring. In addition, a large number of unsaturated zirconium metal sites on the surface of the material might facilitate the post modification [28]. Particularly, the four single strands of the TDN structure were designed and synthesized (Table 1), in which the 5′ ends of the three strands were phosphorylated, and a piece of recognition probe sequence (i.e., the nucleic acid aptamer sequence of CEA) was added to the 5′ end of the fourth strand. Through the self-assembly behavior of DNA based on the Watson–Crick complementary pairing principle, a TDN structure assembled by three phosphorylated vertices was obtained. Reusing the strong interaction of phosphate groups with zirconium ions, the obtained TDN was easily grafted onto the surface of PCN-222 (Fe), which constitutes the final product CEA_apt_-TDN-MOF colloid nanorods. Since the TDN is a rigid structure, a good assembly layer for molecular recognition was formed at the interface of MOFs, which might facilitate the detection of targets. Since the aptamer sequence for CEA recognition was incorporated in the TDN structure, the constructed CEA_apt_-TDN-MOF was potentially a high-performance biosensor. Owing to the iron porphyrin ring, it might exhibit catalytic oxidation activity by using TMB as the chromogenic substrate for the detection of CEA.

**Figure 1:**
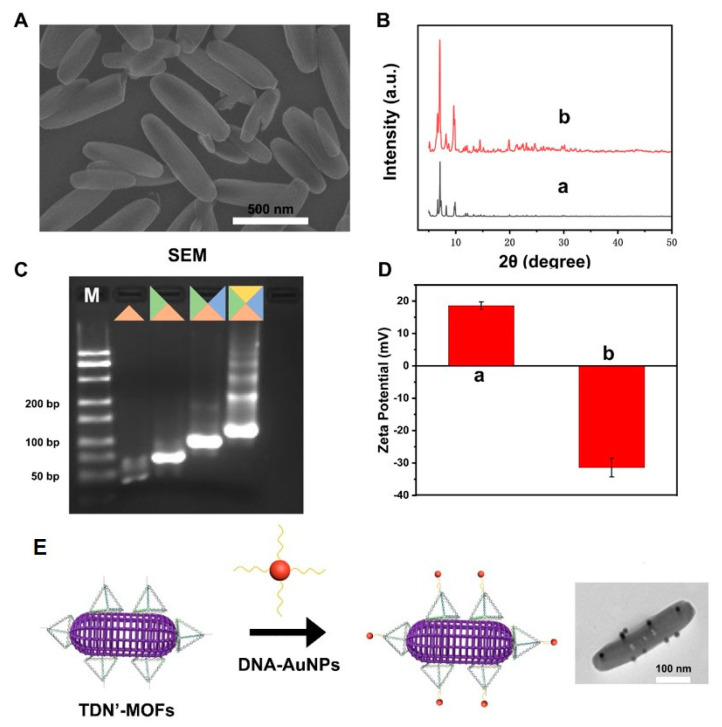
(**A**) Scanning electron microscope (SEM) imaging patterns of PCN-222 (Fe). (**B**) Powder X-ray diffraction patterns of a single crystal-simulated PCN-222 (Fe) structure (a) and a synthesized PCN-222 (Fe) material (b). (**C**) Agarose gel electrophoresis verification of TDN structures. (**D**) The zeta potential of PCN-222 (Fe) material (a) as well as the synthesized CEA_apt_-TDN-MOF (b). (**E**) Schematic representation of molecular recognition event that occurred between DNA-AuNPs (guest) and the newly prepared TDN’-MOFs (host), with a transmission electron microscope (TEM) image.

First of all, the scanning electron microscope (SEM) was used to observe the structure and morphology of the MOF material PCN-222 (Fe), as shown in Figure 1A. Unlike the previously synthesized sphere MOF [29], this time, the obtained MOF material had a rod-like structure with a length of 400–600 nm and a width of about 100 nm. To further verify the ingredients of PCN-222 (Fe), the samples were analyzed by powder X-ray diffraction (PXRD, Figure 1B). The results show that the PXRD spectra of the PCN-222 (Fe) had sharp characteristic diffraction peaks and were highly similar to the simulation results of PCN-222 (Fe) single crystals, indicating that the PCN-222 (Fe)-based MOF material was successfully synthesized. In addition, taking advantage of the DNA self-assembly effect, the A, B, and C single-strand DNA and D chains with phosphorylation modification were annealed to form a TDN structure. In order to study the TDN structure, F, G, and H single-strand DNA (which were similar sequences without phosphorylation modification, with respect to A, B, C single-strand DNA) and D chains were used for agarose gel electrophoresis (Figure 1C). The first lane is a DNA marker band, and the other lanes are a single-stranded DNA of D, a DNA assembled by two chains of D, a DNA assembled by three chains of D/F/G, and a TDN structure assembled by four chains of D/F/G/H. With the increasing of strands, the electrophoresis rate of the nucleic acid structure decreased gradually, indicating that a TDN structure was successfully obtained. Then, using the strong interaction between phosphate and zirconium ions, PCN-222 (Fe) was incubated with phosphorylated TDN to prepare CEA_apt_-TDN-MOF. In Figure 1D, the charge change before and after TDN modification was clearly observed by zeta potential analysis. There was a positive charge on the surface of PCN-222 (Fe) at the beginning, and the zeta potential was +18.6 mV. After the TDN functionalization, it shows that the charge changes to negative value of −31.4 mV, indicating that TDN might be successfully grafted on the surface of PCN-222 (Fe).

In order to further explore the successful modification of TDN on the MOF nanorods, the D chain was replaced by an E chain with a large number of T base sequences at the end, and we assembled a new tetrahedral structure of TDN’ with four single strands of A, B, C, and E DNA. At the same time, the I chain with a multi-A base sequence was grafted on gold nanoparticles with a size of about 13 nm to prepare DNA-AuNPs. Using the complementary pairing of the sequence of A-T bases, a strong molecular recognition event could occur between DNA-AuNPs and the newly prepared TDN’-MOFs. As shown in Figure 1E, the interaction between the two was directly observed by transmission electron microscope (TEM). A large number of AuNPs surround the surface of TDN’-MOFs, indicating a strong molecular recognition. These results fully demonstrate that a DNA tetrahedron was successfully grafted on the surface of PCN-222 (Fe), that is, the CEA_apt_-TDN-MOF was successfully prepared. Moreover, the prepared CEA_apt_-TDN-MOF might efficiently detect its target molecule.

### 2.2. Horseradish Peroxidase Mimic Activity Study

**Figure 2:**
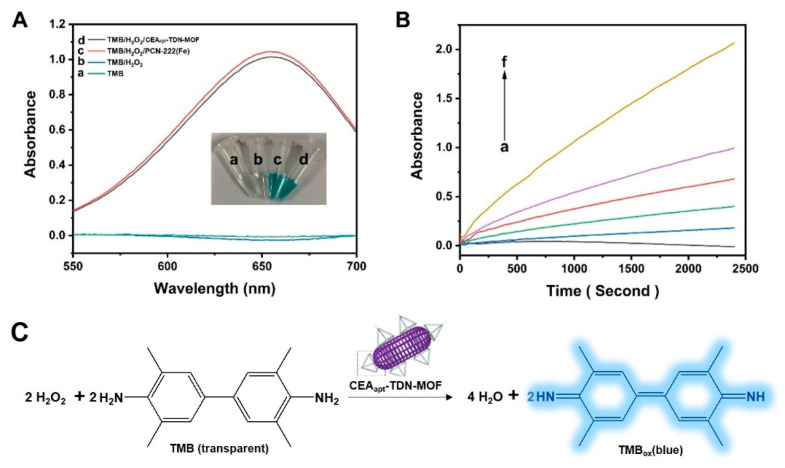
(**A**) UV absorption spectra of the reaction solutions of four groups including TMB, TMB/H_2_O_2_, TMB/H_2_O_2_/PCN-222 (Fe), and TMB/H_2_O_2_/CEA_apt_-TDN-MOF. The embedded plot is a color consequence plot of the responses. (**B**) Kinetic plots of TMB solution catalysis by using different concentrations of CEA_apt_-TDN-MOF. The measurement wavelength was 652 nm. (**C**) Schematic illustration of TMB oxidation reaction, which was catalyzed by CEA_apt_-TDN-MOF, thus resulting in color change from transparent to blue.

To achieve the application purpose of serum tests, the horseradish peroxidase mimic activity study was designed to be carried out in 1 × PBS containing 0.05% Tween-20 (PBST, pH ~7), where the CEA_apt_-TDN-MOF remained 27% of the maximum activity (Figure S2). To investigate the horseradish peroxidase mimicking activity of our CEA_apt_-TDN-MOF, H_2_O_2_ was added to the experimental system to catalyze the oxidation of TMB substrate, which resulted in the color change of the solution. Figure 2A shows the UV absorption spectra of TMB, TMB/H_2_O_2_, TMB/H_2_O_2_/PCN-222 (Fe), and TMB/H_2_O_2_/CEA_apt_-TDN-MOF solutions and the corresponding color response results. There was almost no color change in the solutions of TMB and TMB/H_2_O_2_ (a and b), but there was an obvious blue change in the solution after the addition of PCN-222 (Fe) (c), indicating that the PCN-222 (Fe) had a very good catalytic oxidation activity. Under the same reaction conditions, the reaction solution of CEA_apt_-TDN-MOF (d) also changed obviously blue. To intuitively compare the differences between each group, the UV absorption spectra of TMB in each group’s solution was recorded at a wavelength range of 550–700 nm (with a peak at 652 nm), and the results remained in accordance with the color reaction changes. The group a and group b spectra were similar, showing no UV absorption peak at this wavelength range. Meanwhile, both group c and group d had very high peaks, indicating the high completion of catalytic reaction. As illustrated in Figure 2C, the TMB oxidation reaction was catalyzed by the CEA_apt_-TDN-MOF via Fenton reaction: Fe^2+^ + H_2_O_2_ → Fe^3+^ + HO·+ OH^−^. Moreover, group d had a slightly lower peak than group c but almost negligible, indicating that TDN functionalization had very little effect on the catalytic activity of PCN-222 (Fe), which could effectively guarantee the feasibility of the experiment. In addition, the catalytic ability of CEA_apt_-TDN-MOF was studied by recording the time-varying absorbance curve of TMB at 652 nm. As shown in Figure 2B, the kinetic curve showed that the catalytic ability of CEA_apt_-TDN-MOF increased with the increase of concentration, and the corresponding concentrations of each curve were 0, 6.25, 12.5, 25, 35, and 50 μg/mL (a to f), respectively. Therefore, the catalytic performance of the system could be achieved by adjusting the concentration of CEA_apt_-TDN-MOF in practical applications. Moreover, it was found that this CEA_apt_-TDN-MOF exhibited superior catalytic effect compared with nature horseradish peroxidase (Figure S3), indicating the great potential of this detection system.

### 2.3. Optimization of Experimental Conditions

**Figure 3:**
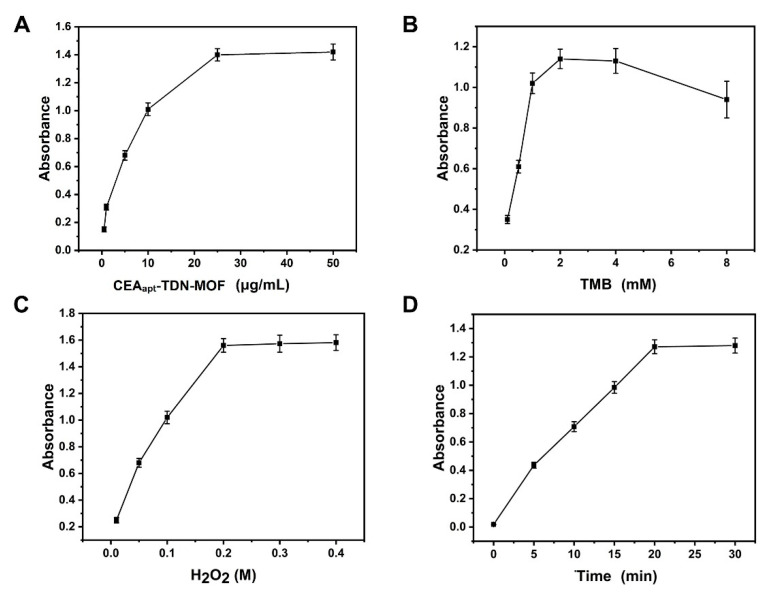
Optimization studies of different experimental conditions for colorimetric detection of CEA. (**A**) Concentration investigation of CEA_apt_-TDN-MOF. (**B**) Concentration investigation of TMB. (**C**) Concentration investigation of H_2_O_2_. (**D**) Detection time investigation. Error values were obtained from triplicate experiments.

First, phosphorylated single-strand DNA (chain J) containing a CEA aptamer was grafted on the surface of PCN-222 (Fe) to prepare CEA_apt_-MOF for the colorimetric sensing of CEA. The results show that the signal response value of CEA_apt_-TDN-MOF was higher than that of CEA_apt_-MOF under the same conditions (CEA concentration is 20 ng/mL), which was probably because the rigid structure of TDN could effectively promote the molecular recognition between the aptamer sequence and target protein, while the flexible single-strand structure might adhere to the surface of the MOF material and reduce the binding efficiency by 35% [30]. Moreover, the detection performance had no change during one year in PBS at 37 °C, indicating the excellent stability of CEA_apt_-TDN-MOF with respect to CEA_apt_-MOF (showing an obvious downward trend in detection ability from the sixth month) (Figure S1). Meanwhile, the shelf-life of the DNA oligo in PBS at 37 °C is only 6 weeks [31]. Therefore, the use of rigid TDN is very necessary for establishing a stable detection system. Then, the CEA aptamer was integrated into DNA tetrahedron so as to construct a colorimetric biosensor based on the molecular recognition ability and oxidation reaction catalytic property. In order to obtain good detection performance, the experimental conditions were optimized according to the response results of the color signal. First of all, the concentration of CEA_apt_-TDN-MOF was optimized, since the concentration of the material directly determined the catalytic performance and the detection effect. As shown in Figure 3A, when the concentration of CEA_apt_-TDN-MOF increased continuously, the colorimetric signal increased all the time and tended to be stable when the concentration reached 25 μg/mL, so this concentration was selected for detection use. Then, the concentrations of chromogenic substrate TMB and oxidant H_2_O_2_ were optimized, respectively. As shown in Figure 3B,C, the detection effect was optimal when the concentrations of TMB and H_2_O were 2 mM and 0.2 M, respectively. Furthermore, according to the results of Figure 3D, the detection time should be 20 min. Under the optimized conditions, CEA detection was carried out.

### 2.4. Assay Performance Analysis

**Figure 4:**
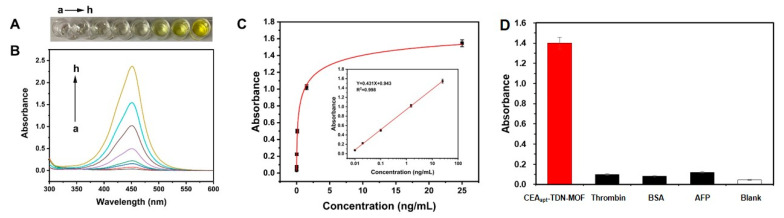
(**A**) Color response and (**B**) UV absorption spectra obtained by experimental detection of CEA at different concentrations. (**C**) The peak values in (**B**) are plotted versus CEA concentration. The inset was the linear plots of peak values versus the logarithm of CEA concentration. Error values were obtained from triplicate experiments. (**D**) Specificity experimental analysis of this colorimetric sensor.

After optimization of the conditions, the designed colorimetric sensing method was applied to detect different concentrations of CEA, and the results after stopping the reaction with 1 M sulfuric acid are shown in Figure 4A. The color response gradually became deeper with increasing concentrations of CEA (the concentrations were 0, 0.005, 0.01, 0.02, 0.1, 1.5, 25, and 50 ng/mL, respectively). At the same time, the corresponding UV absorption spectra were also measured, and the results are shown in Figure 4B. By plotting the UV absorption value of each group at 450 nm versus the logarithm of CEA concentration, it was found that the CEA_apt_-TDN-MOF exhibited a good linear detection range of 0.01–25 ng/mL, and the detection limit was 3.3 pg/mL (calculated according to the equation: D = 3σ/k, where σ is the relative standard deviation of the blank sample, k is the slope of the calibration line, Figure 4C) [32], showing great potential for CEA detection in human serum. In order to further study the specificity of the sensing system, different proteins such as thrombin, bovine serum albumin (BSA), and alpha-fetoprotein (AFP) were used as controls. It was found that the CEA_apt_-TDN-MOF exhibited a negligible detection signal toward control proteins (i.e., thrombin, BSA, AFP) even at the concentration of 100 ng/mL. These results demonstrate that our colorimetric sensing system has very high sensitivity and specificity (Figure 4D).

**Figure 5:**
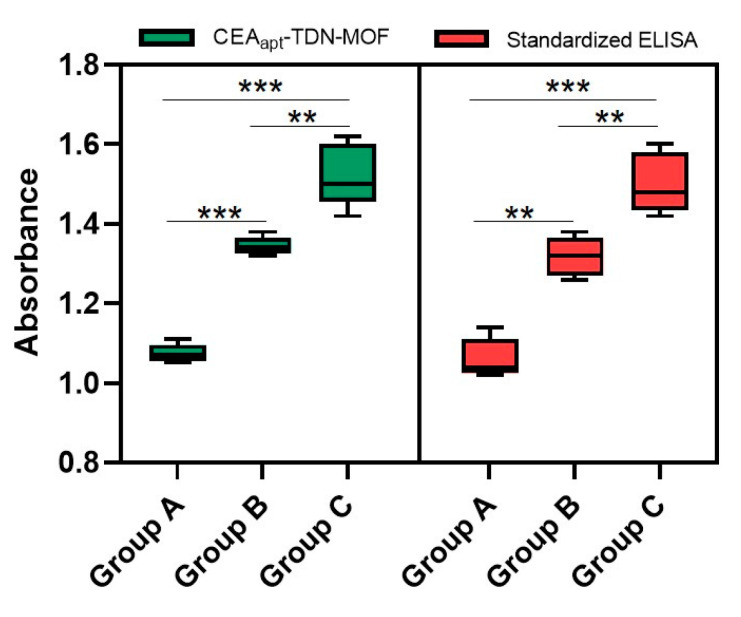
Boxplots showing the distribution of CEA concentrations in serum samples from healthy individuals (group A), patients with early-stage (group B) colon cancer, and patients with advanced-stage (group C) colon cancer. Experiments were carried out using the CEA_apt_-TDN-MOF and standardized ELISA, respectively. The statistical significance of observed differences was determined by *t*-test. ** *p* < 0.01 and *** *p* < 0.001.

To demonstrate the potential of the CEA_apt_-TDN-MOF for clinical uses, the detection of CEA in human serum was carried out by using CEA_apt_-TDN-MOF and confirmed by standardized ELISA. According to clinical studies, the level of CEA in serum is closely related to the occurrence of colon cancer [33,34]. Therefore, the serum samples from healthy individuals, patients with early colon cancer, and patients with advanced colon cancer were used. As shown in Figure 5, the concentration of CEA in serum of patients was significantly higher than that of healthy individuals, and there was also a significant difference in the level of CEA between patients with early and late-stage colon cancer. On the whole, with the improvement of the malignant degree of colon cancer, the content of CEA in blood also increased, indicating that our method has a broad application prospect in tumor diagnosis.

## 3. Conclusions

In this work, we successfully functionalized DNA tetrahedral nanostructures onto PCN-222 (Fe) materials with simulated horseradish peroxidase activity and constructed a colorimetric immune biosensor for the ultra-sensitive detection of CEA. The phosphorylated DNA tetrahedral structure could be effectively fixed on the PCN-222 (Fe) surface through strong coordination with zirconium ions, thus bringing very stable performance. Moreover, the rigid structure of TDN greatly promoted the binding of the aptamer sequence at the end of the tetrahedron to the target. As a consequence, the strong molecular recognition ability and high enzyme catalytic activity ensure that the colorimetric sensing method has very high specificity and sensitivity, and the detection limit is as low as 3.3 pg/mL (superior with respect to other sensors of the same type, Table S1) [3,4,35,36,37,38,39,40,41], even in clinical serum samples, so it has great application potential. In addition, the strategy proposed in this work can be extended to other aptamer sequences, so it has high universality.

## 4. Materials and Methods

### 4.1. Instrumentation

Scanning Electron Eicroscope (SEM, S-3400N II, Hitachi Corporation, Nara, Japan), Transmission Electron Microscopy (TEM, TECNAI G2 F20 S-TWIN, Tokyo, Japan), X-ray Powder Diffractometer (PXRD, D8 Advance, Bruker, Berlin, Germany), Milli-Q ultrapure water purification system (Millipore, Milford, USA), zeta potential analyzer (NanoBrook 90Plus, Brookhaven, Holtsville, USA), UV spectrophotometer (UV-1800, Shimadzu, Kyoto, Japan), and multifunctional microplate reader (M200 Pro, Tecan, Männedorf, Switzerland) were applied.

### 4.2. Reagents

Zirconia dichloride octahydrate and meso-tetra (4-carboxyphenyl) porphine ferric chloride (Fe-TCPP) were purchased from Shanghai Lingwei Reagent Co., Ltd. (Shanghai, China). *N*,*N*-dimethylformamide (DMF) and its chromogenic substrate 3,3′,5,5′-tetramethylbenzidine (TMB) were purchased from Sigma-Aldrich (St. Louis, MI, USA). 1 × PBS (containing 0.05% Tween-20) solution, blocking buffer (BSA), and universal antibody dilutions were purchased from Shanghai SANGON biotech Co., Ltd. (Shanghai, China). Human carcinoembryonic antigen (CEA) and CEA antibody were purchased from Shanghai LingChao Bio Technology Co., Ltd. (Shanghai, China). A Human CEA ELISA Kit (Catalog Number: EHCEA) was purchased from Thermo Fisher (Shanghai, China). Gold nanoparticles (AuNPs) with a diameter of 13 nm were purchased from Jiangsu Shengfeng nanomaterials technology Co., Ltd. (Suzhou, China). The 96-well plates were purchased from Corning (Corning, NY, USA). Water used throughout the experiment was ultrapure water (impedance >18 m Ω•cm, purified from a Milli-Q ultrapure water system). The remaining chemicals were of analytical grade and used without further purification. All oligonucleotide strands were synthesized by the Shanghai bioengineering company and subjected to HPLC purification. DNA sequences are shown in Table 1.

### 4.3. Synthesis of Metal–Organic Framework PCN-222 (Fe)

First, 38 mg of zirconia dichloride octahydrate was dissolved with 6.5 mg of Fe-TCPP in 16.3 mL of DMF and sonicated for 1 min to mix well. Then, 0.25 mL of dichloroacetic acid was added into the mixed solution, transferred into a Teflon-lined hydrothermal reaction tank, and tightened to sit in a 180 °C oven for 18 h to heat. After the reaction, the as-synthesized nanocrystals were obtained by the method of centrifugation (10,500 rpm, 10 min), which was immediately followed by centrifugal washing with DMF and ethanol solution to remove the precursor species that did not participate in the reaction, and repeated 3 times. Finally, the solid was dried under vacuum at 80 °C overnight for further use.

### 4.4. Preparation and Characterization of TDN Structures

The preparation process of TDN with aptamer sequences was described as following: four single-stranded DNAs (A, B, C, D) were mixed in TM buffer (10 mm Tris HCl, 50 mM MgCl_2_, pH 8.0) at equal ratios. Then, the solution was heated to 95 °C and re-cooled to 4 °C to complete the renaturation process. In addition, the preparation of TDN-bearing poly-T sequences (four single-stranded DNAs, A, B, C, E) was similar to the procedure described above. Finally, the as-prepared two tetrahedron structures were stored at 4 °C for further use.

Agarose gel electrophoresis: the buffer conditions were 1 × TBE, 3% agarose gel, electrophoresis at 100 V for 40 min, and the TDN (four single-stranded DNA of D, F, G, H) concentration was 1 μM.

### 4.5. Preparation of TDN-Functionalized Modified PCN-222 (Fe): CEA_apt_-TDN-MOF Colloid Nanorods

First, 0.2 mL solution containing TDN (10 μM) was added to 2 mg (783 nmol) PCN-222 (Fe) nanorods and incubated on a shaker for 4 h. During this period, the NaCl solution was added slowly to reduce the electrostatic repulsion, and the final concentration was 0.5 M. Then, the excess nucleic acid structure was removed by centrifugal washing (10,000 rpm, 15 min), and the operation was repeated three times. Finally, it was re-dispersed into TM buffer and stored at 4 °C for use.

### 4.6. Preparation of DNA-AuNPs and TDN-MOFs

First, 1 mL of 40 nm diameter gold nanoparticles was taken from the mother liquor into a low-adsorption EP tube, centrifuged for 15 min (16,200× *g*) at 4 °C, and resuspended in 1 mL of 10 nM phosphate (PB) buffer. In addition, 100 μM of thiol-modified DNA sequence (strand J) was activated by incubation with 10 nM of TCEP solution for 1 h. Then, 35 μL of activated DNA was added to the above gold nanoparticles solution (the final concentration was 3 μM) and incubated for 16 h. Afterwards, 9 μL of NaCl solution (2 M) was gradually added to the reaction system, and the final salt concentration was 0.1 M, during which we were gently tapping the wall to avoid aggregation. The DNA-modified gold nanoparticles were centrifuged at 4 °C for 15 min (16,600× *g*) to remove the supernatant and resuscitated with 10 mM PB buffer. Then, 500 nmol PCN-222 (Fe) nanorods decorated with TDN with poly-T sequences were incubated with DNA-modified gold nanoparticles for 2 h to complete the assembly behavior, and finally, DNA-modified gold nanoparticles without the assembly behavior occurring were removed by centrifugation.

### 4.7. Construction of an Immunosensor for the Detection of CEA

First, 100 μL of capture antibody anti-CEA solution (3 μg/mL) was added to each well of a 96-microwell plate and incubated at 4 °C overnight. After the antibody solution was removed, the antibody solution was washed with PBST buffer. Then, 150 μL BSA sealing solution was added to each well. After incubation at 37 °C for 1 h, it was washed 3 times with PBST buffer. Then, each well was incubated with different concentrations of the target protein (CEA) 37 °C for 1 h, the well with only PBST solution was set as the blank control group, and the washing step was repeated three more times. This was immediately followed by the addition of 100 μL of TDN-functionalized modified PCN-222 (Fe) nanorods (25 μg/mL, containing 5 nM PCN-222 (Fe)) to each well, and the wells were sealed with a sealing film and incubated at room temperature for 1 h. Finally, 100 μL of TMB substrate solution (2 mM TMB and 0.2 M H_2_O_2_) was added with gentle shaking for 20 min, after which 50 μL of stop solution (1 M concentrated sulfuric acid) was added to stop the reaction, and the absorbance intensity was measured at 450 nm with a microplate reader. The standardized ELISA test was performed by following the instructions for the product: EHCEA (96 tests) at thermofisher.com, accessed on 18 October 2021.

Human serum samples from colon cancer patients were obtained from the Second Affiliated Hospital of Nanjing, Southeast University. Informed consent was obtained from all cases, and this study was approved by the Scientific and Ethical Committee of Nanjing University and Southeast University. The serum sample detection chamber simply needs to exchange the target solution for a 100 μL serum samples, and the other processes are similar to the above steps.

## Figures and Tables

**Table 1:** DNA tetrahedron motif sequences.

DNA	Sequence (from 5′ to 3′)
Strand A	5′-H_2_PO_3_-TATCACCAGGCAGTTGACAGTGTAGCAAGCTGTAATAGATGCGAGGGTC CAATAC-3′
Strand B	5′-H_2_PO_3_-TCAACTGCCTGGTGATAAAACGACACTACGTGGGAATCTACTATGGCGG CTCTTC-3′
Strand C	5′-H_2_PO_3_-TTCAGACTTAGGAATGTGCTTCCCACGTAGTGTCGTTTGTATTGGACCC TCGCAT-3′
Strand D	5′-ATACCAGCTTATTCAATTTTTTTTACATTCCTAAGTCTGAAACATTACAGCTTGC TACACGAGAAGAGCCGCCATAGTA-3
Strand E	5′-TTTTTTTTTTTTTTTTTTTTTACATTCCTAAGTCTGAAACATTACAGCTTGC TACACGAGAAGAGCCGCCATAGTA-3′
Strand F	5′-TATCACCAGGCAGTTGACAGTGTAGCAAGCTGTAATAGATGCGAGGGTC CAATAC-3′
Strand G	5′-TCAACTGCCTGGTGATAAAACGACACTACGTGGGAATCTACTATGGCGG CTCTTC-3′
Strand H	5′-TTCAGACTTAGGAATGTGCTTCCCACGTAGTGTCGTTTGTATTGGACCC TCGCAT-3′
Strand I	5′-SH-AAAAAAAAAAAAAAAAAAAAAAA-3′
Strand J	5′-H_2_PO_3_-TTTTTTTATACCAGCTTATTCAA-3′

## Supplementary Materials

The following are available online at https://www.mdpi.com/article/10.3390/gels7040181/s1, Figure S1. Detection performance of the CEA at 20 ng/mL by CEAapt-TDN-MOFs or CEAapt-MOFs at 25 μg/mL (A) and the record during one year (B). The experiment was performed three times. Figure S2. Detection performance of 25 μg/mL CEAapt-TDN-MOFs toward 20 ng/mL CEA at pH ranging from 1 to 11. The experiment was performed twice to provide mean values as results. Figure S3. Lineweaver–Burk plot of catalytic activity in the presence of CEAapt-TDN-MOFs (▪); HRP (□). Insert table: Km and Vmax values obtained by analysis of Lineweaver–Burk plots. Table S1. Comparison of CEA testing methods in the past five years.
