# Supplementary material for: Stable DNA Aptamer–Metal–Organic Framework as Horseradish Peroxidase Mimic for Ultra-Sensitive Detection of Carcinoembryonic Antigen in Serum
Source: Gels. 2021 Oct 25;7(4):181. doi: 10.3390/gels7040181 (PMC8628696; doi:10.3390/gels7040181)
Supplement: Supplementary file 1 [file gels-07-00181-s001.zip › gels-1384750-supplementary.pdf]

gels

MDPI

Supplementary Information

Stable DNA Aptamer–Metal Organic Framework as Horseradish Peroxidase Mimic for Ultra-Sensitive Detection of Carcinoembryonic Antigen in Serum

Lingjun Sha [1,†], Mingcong Zhu [2,†], Fuqing Lin [3], Xiaomeng Yu [1], Langjian Dong [1], Licheng Wu [2], Rong Ding [2], Shuai Wu [4,*] and Jingjing Xu [1,2,*]

1 Center for Molecular Recognition and Biosensing, School of Life Sciences, Shanghai University, Shanghai 200444, China; lingjun_sha21@163.com (L.S.); xiaomeng_yu2021@163.com (X.Y.); langjian_dong@163.com (L.D.)
2 Sino-European School of Technology of Shanghai University, Shanghai University, Shanghai 200444, China; cytheriacc@126.com (M.Z.); wulich1997@gmail.com (L.W.); Rongding_2021@163.com (R.D.)
3 School of Basic Medical Sciences, Fudan University, Shanghai 200433, China; 2030150160@fdu.edu.cn
4 State Key Laboratory of Pharmaceutical Biotechnology, School of Life Sciences, Nanjing University, Nanjing 210023, China
* Correspondence: shwu@njmu.edu.cn (S.W.); jingjing_xu@shu.edu.cn (J.X.)
† These authors contributed equally to this work.

Colloid stability study by detection performance record

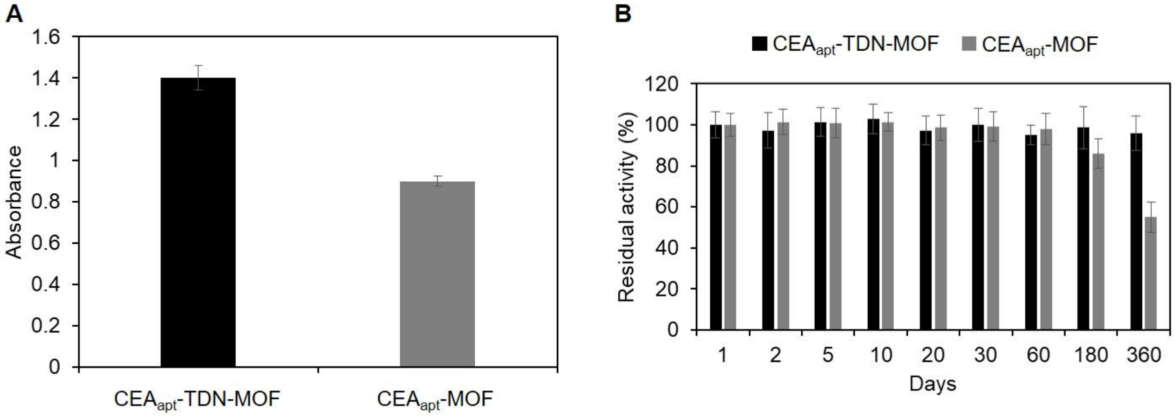

Figure S1. Detection performance of the CEA at 20 ng/mL by CEA$_{apt}$-TDN-MOFs or CEA$_{apt}$-MOFs at 25 μg/mL (A) and the record during one year (B). The experiment was performed three times.

Detection performance study at different pH

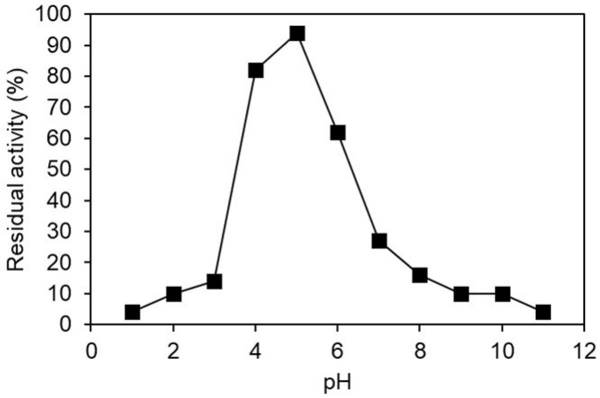

Figure S2. Detection performance of 25 μg/mL CEA_{apt}-TDN-MOFs toward 20 ng/mL CEA at pH ranging from 1 to 11. The experiment was performed twice to provide mean values as results.

Detection performance study at different pH

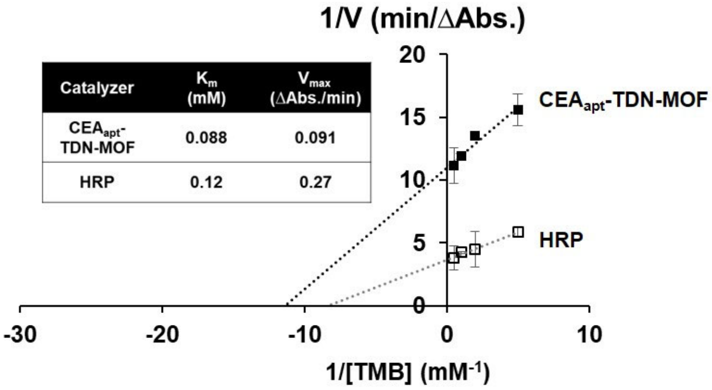

Figure S3. Lineweaver-Burk plot of catalytic activity in the presence of 5 nM CEA_{apt}-TDN-MOFs (■); 5 nM HRP (□). Insert table: K_m and V_{max} values obtained by analysis of Lineweaver-Burk plots.

CEA detection performance study using different sensors

Table S1. Comparison of CEA testing methods in the past five years.

Detection method	Limit of detection	Linear detection range	Reference
Photoelectric sensing	17.4 pg/mL.	0.04 – 60 ng/mL	[1, 2]
	32 pg/mL	0.1 – 300 ng/mL	
Fluorescence sensing	6.7 pg/mL	0.05 – 20 ng/mL	[3, 4]
	41 pg/mL	0.1 – 200 ng/mL	
Pressure sensing	167 pg/ml	0.5 – 60 ng/mL	[5, 6, 7, 8]
	87 pg/ml	0.1 – 40 ng/mL	
	130 pg/mL	0.2 – 60 ng/mL	
	150 ng/mL	0.2 – 80 ng/mL	
Colorimetric sensing	140 pg/mL	0.5 – 15 ng/mL	[9]
**This work**	**3.3 pg/mL**	**0.01 – 25 ng/mL**	

References

[1] Zhang, K.; Lv, S.; Tang, D. A 3D printing-based portable photoelectrochemical sensing device using a digital multimeter. *Analyst* **2019**, *144*, 5389–5393.

[2] Lv, S.; Zhang, K.; Zhu, L.; Tang, D. ZIF-8-Assisted NaYF4:Yb,Tm@ZnO Converter with Exonuclease III-Powered DNA Walker for Near-Infrared Light Responsive Biosensor. *Anal. Chem.* **2020**, *92*, 1470–1476.

[3] Qiu, Z.; Shu, J.; Tang, D. Bioresponsive Release System for Visual Fluorescence Detection of Carcinoembryonic Antigen from Mesoporous Silica Nanocontainers Mediated Optical Color on Quantum Dot-Enzyme-Impregnated Paper. *Anal. Chem.* **2017**, *89*, 5152–5160.

[4] Lv, S.; Tang, Y.; Zhang, K.; Tang, D. Wet NH3-Triggered NH2-MIL-125(Ti) Structural Switch for Visible Fluorescence Immunoassay Impregnated on Paper. *Anal. Chem.* **2018**, *90*, 14121–14125.

[5] Yu, Z.; Tang, Y.; Cai, G.; Ren, R.; Tang, D. Paper Electrode-Based Flexible Pressure Sensor for Point-of-Care Immuno-assay with Digital Multimeter. *Anal. Chem.* **2019**, *91*, 1222–1226.

[6] Yu, Z.; Cai, G.; Tong, P.; Tang, D. Saw-Toothed Microstructure-Based Flexible Pressure Sensor as the Signal Readout for Point-of-Care Immunoassay. *ACS Sens.* **2019**, *4*, 2272–2276.

[7] Yu, Z.; Cai, G.; Liu, X.; Tang, D. Platinum Nanozyme-Triggered Pressure-Based Immunoassay Using a Three-Dimensional Polypyrrole Foam-Based Flexible Pressure Sensor. *ACS Appl. Mater. Interfaces* **2020**, *12*, 40133–40140.

[8] Huang, L.; Yu, Z.; Chen, J.; Tang, D. Pressure-Based Bioassay Perceived by a Flexible Pressure Sensor with Synergistic Enhancement of the Photothermal Effect. *ACS Appl. Bio Mater.* **2020**, *3*, 9156–9163.

[9] Gao, Z.; Shao, S.; Gao, W.; Tang, D.; Tang, D.; Zou, S.; Kim, M.J.; Xia, X. Morphology-Invariant Metallic Nanoparticles with Tunable Plasmonic Properties. *ACS Nano* **2021**, *15*, 2428–2438.
